# *e* MolFrag2 and *e* Synth2: Sound Fragment-Based Molecular Decomposition and Reconstruction

**DOI:** 10.1021/acs.jcim.6c01714

**Published:** 2026-10-13

**Authors:** Adam Bess, Sean Rowland, Chris Alvin, Meisam Adibifard, Supratik Mukhopadhyay

**Affiliations:** † Department of Computer Science, 5779 Louisiana State University, Baton Rouge, Louisiana 70803, United States; ‡ Department of Computer Science, 3628 Furman University, Greenville, South Carolina 29613, United States; § Department of Environmental Sciences, 5779 Louisiana State University, Baton Rouge, Louisiana 70803, United States

## Abstract

We present *e* MolFrag2 and *e* Synth2, enhanced successors to *e* MolFrag and *e* Synth for fragment-based molecular decomposition and reconstruction. *e* MolFrag2 is a complete reimplementation of *e* MolFrag in Python, using RDKit and graph-based molecular representations to preserve connectivity, identify free atoms, combine adjacent linkers, and avoid aromaticity ambiguities that impair downstream reconstruction. *e* Synth2 generalizes fragment recombination by removing hard-coded molecular-property filters, supporting multiple bonds to a single brick atom, and adding a targeted reconstruction mode for soundness validation. Across 36,534 nonredundant DUD-E active compounds, the *e* MolFrag2 → *e* Synth2 workflow reconstructed every input molecule exactly (Tanimoto coefficient, *TC* = 1.0), whereas the original *e* MolFrag→ *e* Synth workflow reconstructed 51 of the 100 molecules in a benchmark subset. The upgraded workflow also produced broader recombinant chemical spaces, including higher entropy in a four-molecule diversity analysis (2.78 versus 2.70). Both tools are open-source, platform-agnostic, and available for academic and commercial use.

## Graphical abstract

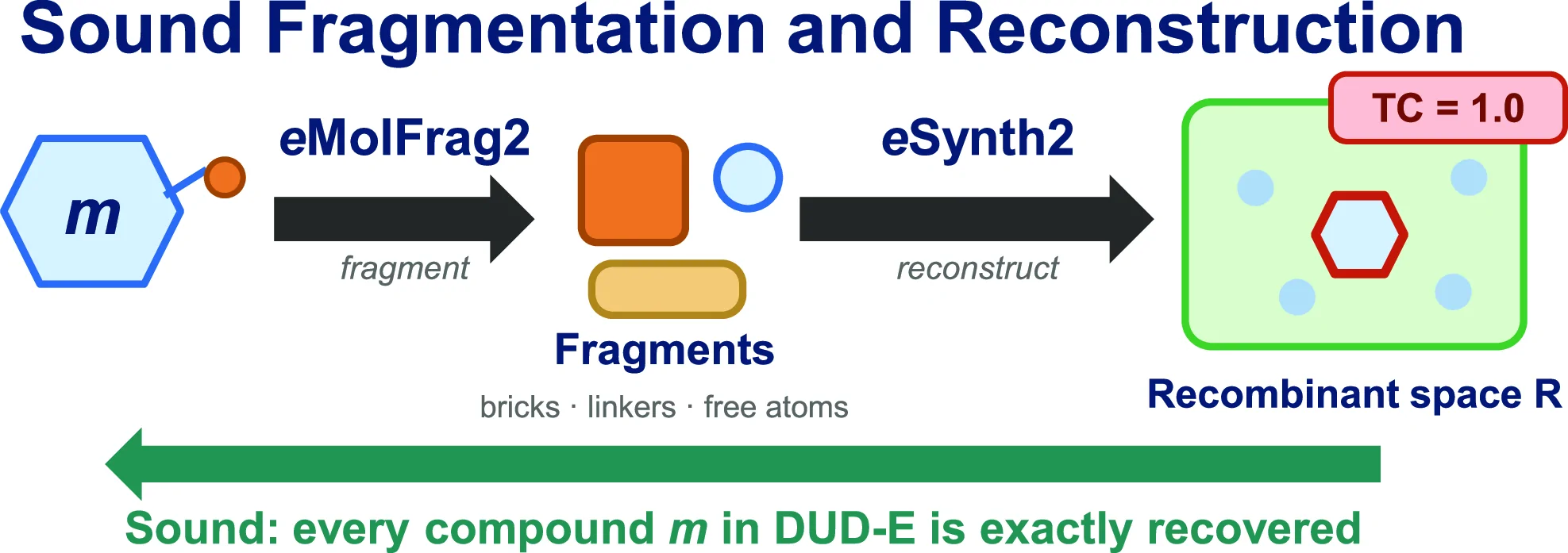

## Introduction

Fragment-based drug design (FBDD)^1^ identifies small-molecule fragments that bind biological targets and uses those fragments as starting points for lead discovery. Computational FBDD workflows depend on two linked capabilities: the reliable decomposition of molecules into chemically meaningful fragments and the reliable recombination of those fragments into searchable chemical spaces. Fragmentation tools such as RECAP,^2^ BRICS,^3^ and matched molecular pair analysis (MMPA)^4^ are widely used for decomposition and have found broad application in virtual screening,^5^ scaffold-hopping,^6^ and AI-guided generative molecular design.^7,8^ However, these tools were designed for fragmentation and structural analysis rather than reconstruction: none provides a native mechanism for recovering the original molecule from its fragments, and none guarantees that a fragmented molecule can be exactly reconstructed from its outputs. This limitation has practical consequences. When fragmentation does not preserve enough connectivity information to reconstruct the original molecule exactly, active compounds can be missed in virtual screening, fragment libraries used to train generative models can carry incomplete or ambiguous bonding information, and fragment-based pipelines cannot be benchmarked reproducibly. A sound fragmentation and reconstruction workflow, one in which every fragmented molecule is exactly recoverable from its fragments, is therefore a prerequisite for reproducible chemical space exploration, generative molecular design, and fragment-based drug discovery. The original *e* MolFrag and *e* Synth tools helped establish this workflow,^9,10^ but practical use revealed limitations in reconstruction accuracy, fragment classification, aromatic bond handling, input flexibility, and long-term maintainability.

Here, we describe *e* MolFrag2 and *e* Synth2, an updated software pair for fragment-based molecular decomposition and reconstruction. The major advances are technical and usability-focused: *e* MolFrag2 is a complete reimplementation of the fragmentation engine in Python using RDKit^11^ and graph-based operations, while *e* Synth2 enhances the original construction engine by removing hard-coded assumptions about drug-likeness and fragment connectivity. Together, the tools provide an end-to-end workflow in which molecules are fragmented, annotated with bond- and atom-type connectivity metadata, and reconstructed into recombinant chemical spaces.

Soundness is the primary scientific requirement for such a workflow, and Figure 1 shows the procedure by which we establish it. In our benchmark, *e* MolFrag2 → *e* Synth2 reconstructed every one of the 36,534 tested active compounds from the Directory of Useful Decoys, Enhanced (DUD-E) database^12^ at a Tanimoto coefficient (TC) of 1.0. This result establishes the updated workflow as a reliable basis for chemical space exploration, virtual screening, and software-assisted fragment-based design.

**Figure 1:**
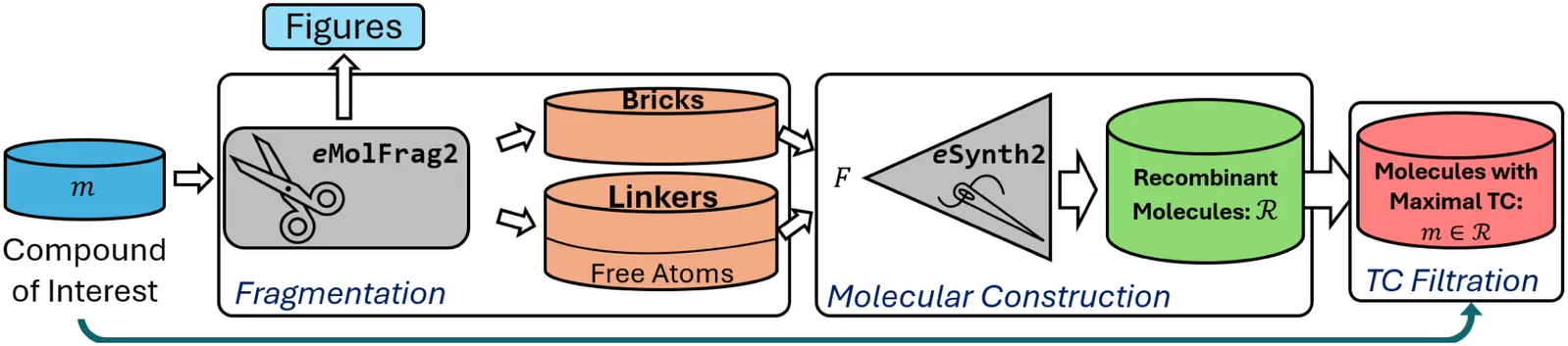
Soundness validation procedure for the updated *e* MolFrag2 → *e* Synth2 workflow. A molecule *m* is fragmented into *F* by *e* MolFrag2, reconstructed into recombinant molecules R by *e* Synth2, and validated by testing whether *m* is present in R with *TC* = 1.0.

## Software Description

*e* MolFrag2 and *e* Synth2 operate as paired workflows. A molecule of interest is first passed to *e* MolFrag2, which decomposes it into a set of chemically meaningful fragments classified as bricks, linkers, and free atoms. These fragments are written to SDF files along with embedded metadata describing the bond types, atom types, and connectivity at each severed connection. *e* Synth2 then reads these files and systematically recombines the fragments, joining compatible connection points to construct a recombinant chemical space. In practice, *e* MolFrag2 is run across a library of molecules, and the resulting fragments are pooled before *e* Synth2 recombines them, so that connection points are drawn from a broader and more diverse fragment set. The single-molecule case used in our soundness validation is a special case of this more general usage. The two tools are sound across all 36,534 nonredundant DUD-E active compounds, as demonstrated in the Validation and Benchmarking section.

### *e* MolFrag2: Molecular Fragmentation

*e* MolFrag2 is a complete reimplementation of *e* MolFrag; no source code from the original implementation was reused. Where *e* MolFrag relied heavily on file and string manipulation, *e* MolFrag2 uses RDKit molecule objects and graph-based analysis through Python’s NetworkX^13^ library. This approach reduces implementation complexity, improves maintainability, and makes it possible to reason directly over molecular connectivity.

#### Free Atoms and Linkers

Both *e* MolFrag and *e* MolFrag2 decompose molecules using a modified BRICS procedure^3^ into fragments termed bricks and linkers. Previously,^9,10^ we classified bricks as rigid sections of the molecular graph consisting of at least 4 nonhydrogen atoms, and linkers as more flexible substructures consisting of 2 or 3 atoms. In the original workflow, single-atom fragments and two- or three-atom fragments were not always classified correctly as linkers; *e* MolFrag instead labeled many of them as bricks. *e* MolFrag2 formally defines single-atom connective fragments as *free atoms*, treats them as a special linker subtype, and records them explicitly in the output files. Recording free atoms explicitly serves two purposes: a free atom may be essential for exact reconstruction, and users can exclude free atoms to construct smaller or more constrained chemical spaces.

#### Linker Combination

*e* MolFrag2 combines adjacent small linkers into larger functional linker units when the graph topology supports this. For example, BRICS bond breaking can produce a chain of small linker fragments in cefmetazole; *e* MolFrag2 represents the same region as a single larger linker (Figure 2(b)). Combining adjacent linkers reduces unnecessary fragmentation and produces fragments that better preserve the local chemical context. In the DUD-E benchmark, linker atom count distributions from *e* MolFrag2 showed a greater spread than those from *e* MolFrag (standard deviation 1.52 versus 0.50), consistent with *e* MolFrag2 successfully combining adjacent linkers.

**Figure 2:**
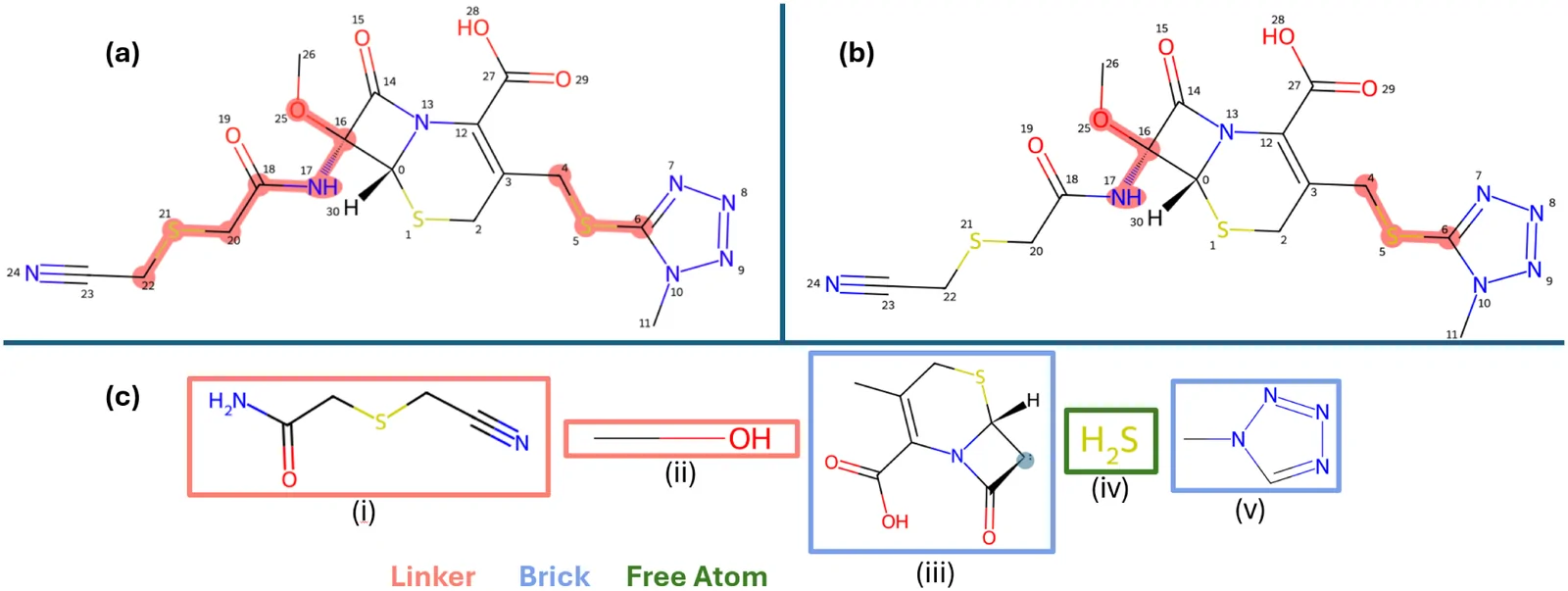
Cefmetazole (DB00274 ^14^) molecular structure and its constituent fragments produced by *e* MolFrag2. (a) Annotated with all bonds that would be snipped by the BRICS algorithm. (b) Annotated with all bonds that would be snipped by *e* MolFrag2; fragment border colors in (c) correspond to the highlighted bond colors in (b). (c) The five unique fragments produced by *e* MolFrag2, ordered left to right to reflect their positional occurrence in the molecule shown in (b): (i) linker, (ii) linker, (iii) brick, (iv) free atom, (v) brick.

#### Connectivity and Superfragments

*e* MolFrag2 records the original bond type, corresponding atom types, and fragment class for each severed connection. For molecules containing fragments that are chemically identical (*TC* = 1.0) but expose different connection points, *e* MolFrag2 can define a *superfragment*. This is a single representative fragment constructed to encompass the union of all bonding patterns exposed across equivalent fragments. As a result, no valid connection point is lost when only one representative is retained for the reconstruction. A worked example of superfragment construction for the DUD-E molecule aldr-102 is provided in the Supporting Information, including Figure S1.

When outputting fragments, *e* MolFrag2 may encounter multiple *TC* = 1.0 equiv fragments and output only one representative; that representative may not have the richest connectivity information. Even when all equivalent fragments are passed to *e* Synth2, the processing order matters: a fragment with lower connectivity may be considered first, causing a richer fragment to be filtered out as redundant and reconstruction at *TC* = 1.0 to fail.

#### Radical Atom Representation

During fragment extraction, *e* MolFrag2 explicitly tracks radical electrons that arise from bond cleavage. Before removing atoms from the original molecule, the tool counts the number of bonds (*N*_*b*,*cut*_) severed from each fragment atom. After excision, atoms with nonzero *N*_*b*,*cut*_ values have their radical electron counts updated accordingly. These radical atoms are preserved in the output SDF file using the standard MDL MOL radical specification (M RAD records following the bond block). This explicit representation keeps fragment valence states unambiguous and helps downstream tools correctly interpret the chemical states of connection points.

#### Aromaticity Handling

RDKit’s kekulization^11^ can alter aromatic bond representations during molecule manipulation. Because ambiguous aromatic bonds caused reconstruction failures in the original workflow, *e* MolFrag2 imports aromatic systems using explicit single and double bonds and prevents subsequent kekulization from changing the representation needed by *e* Synth2. This design makes fragment metadata unambiguous during reconstruction.

#### Input, Output, and Software Quality

*e* MolFrag2 accepts RDKit-supported molecular formats, including MOL2, SDF, SMILES,^15^ and MOL, rather than being limited to MOL2 input. It outputs SDF files containing fragment structures and embedded metadata for bond type, atom type, and fragment classification. *e* Synth2 outputs reconstructed molecules as SMILES strings; three-dimensional coordinate information from the input fragments is not carried through to the reconstructed molecules. Users requiring 3D conformers of reconstructed molecules should apply a dedicated conformer generation tool such as RDKit’s EmbedMolecule or OpenBabel^16^ as a postprocessing step. The codebase is smaller and more thoroughly documented than *e* MolFrag (1,760 source lines versus 3,432; 1,504 comment lines versus 523), and it includes automated tests through GitHub Actions.

### *e* Synth2: Molecular Reconstruction

*e* Synth2 constructs recombinant molecules from the SDF fragment output of *e* MolFrag2. It reads the connectivity metadata associated with each fragment, joins compatible connection points, and saturates open valences with hydrogen atoms as molecules are grown fragment-by-fragment. The updated implementation preserves the graph-based design objective of *e* Synth while removing assumptions that restricted the original tool.

#### Multiple Bonds to a Single-Brick Atom

The original *e* Synth assumed that only linker atoms could participate in multiple BRICS-derived bonds. However, molecules such as cefmetazole contain brick atoms that require multiple fragment connections for exact reconstruction (Figure 2(c)). *e* Synth2 supports these cases directly, eliminating a reconstruction failure mode in the original workflow.

#### Removal of Restrictive Filters

*e* Synth included hard-coded filters motivated by Lipinski’s Rule of Five^17^ to reduce the size of the generated spaces. Although useful in some drug discovery settings, these filters exclude many bioactive molecules and chemical families; for example, terpene molecular weights can exceed conventional drug-like limits.^18,19^ *e* Synth2 removes these filters from the core construction algorithm so that users can decide which molecular-property filters are appropriate for a given study.

#### Soundness Verification with the Unique-Build Option

*e* Synth2 provides a unique build option that accelerates soundness verification. When this option is enabled, each fragment from an input molecule is used exactly once, except for superfragments that represent multiple chemically equivalent fragments. The option still enumerates superfluous recombinant molecules before reaching the original molecule, but it does so far more efficiently than unconstrained enumeration. It therefore provides a practical test of whether the original molecule is present among the recombinants.

## Usage

The tools are designed to be operating-system-agnostic and have been tested on Linux, macOS, and Windows through WSL2. Core dependencies are Python 3.7 or higher; RDKit 2020.09 or higher; NumPy 1.19 or higher; and NetworkX 2.5 or higher. Plotting and analysis scripts additionally use common Python scientific packages, such as pandas and matplotlib.

A typical workflow begins by fragmenting one or more SDF, MOL2, MOL, or SMILES input files with *e* MolFrag2, producing SDF files for the bricks, linkers, and free atoms. Those files are then supplied to *e* Synth2 to construct recombinant molecules or to run targeted validation against an original molecule. A fragment pool should contain both bricks and linkers, since bricks supply the rigid cores of reconstructed molecules and linkers provide the connections between them; free atoms are required when exact reconstruction of an input molecule is the goal and otherwise serve as single-atom connectors that enlarge the recombinant space. The −hl parameter limits the number of fragments permitted in a reconstructed molecule and is the primary control on output volume and runtime; the reconstruction-throughput subsection below characterizes the generation rate for a pool of 20 fragments at −hl = 10. Because the candidate space grows combinatorially in both pool size and the per-molecule fragment limit, users assembling substantially larger pools should raise −hl incrementally. The workflow is intentionally file-based at the interface level so that fragment libraries can be inspected, filtered, shared, and reused in downstream virtual screening pipelines.

## Validation and Benchmarking

### Data Set and Methodology

Because the recombinant space built from a fragment set is potentially unbounded, we test soundness by membership rather than by exhaustive enumeration. For each molecule *m,* we use *e* MolFrag2 to construct the fragment set *F*, use *e* Synth2 to generate a space of recombinant molecules R from *F*, and determine whether m∈R at *TC* = 1.0 (Figure 1).

We benchmarked the updated workflow on active compounds from the DUD-E database. Originally, the DUD-E database offered 40,490 compounds; we obtained a subset of 36,534 unique active compounds across 102 protein targets by eliminating all redundant molecules using a string-based comparison of canonical SMILES generated by RDKit. RDKit canonical SMILES do not encode stereochemistry, protonation states, or tautomeric form; molecules differing only in these respects were therefore not treated as redundant. We chose DUD-E because it served as the benchmark for the original tools and because its diverse range of protein targets makes it a versatile test set. For direct comparison with the original tools, we also used the test-set100 sample distributed with *e* MolFrag. A quantile–quantile (Q-Q) plot,^20^ which assesses whether two distributions share the same shape by plotting their quantiles against each other, compares total fragment counts between test-set100 and the full DUD-E active set. Alignment along the diagonal identity line indicates a similar distributional form. The resulting plot (Figure S2) indicated that this 100-molecule subset was representative of the full DUD-E active set for the fragmentation statistics considered here.

Soundness was evaluated with TC:^21,22^ a reconstruction was considered exact when the original molecule appeared among the recombinants at *TC* = 1.0. Molecular similarity was computed using RDKit topological fingerprints with default parameters: paths of up to 7 bonds, 2 bits per unique subgraph, and a bit vector length of 2048 bits, with explicit hydrogens included in path enumeration. RDKit topological fingerprints do not encode stereochemistry. The canonical SMILES comparison used above for DUD-E redundancy removal likewise ignores stereochemistry. *e* MolFrag2 does not explicitly strip stereochemical information during fragmentation; therefore, chiral tags present in an input molecule are retained on fragment atoms as an RDKit representation detail. However, our soundness criterion does not test for stereochemical equivalence. Therefore, the *TC* = 1.0 soundness guarantee established in this work applies to constitutional (2D graph) reconstruction and does not, by itself, confirm stereochemical fidelity. Stereochemistry-aware validation is a direction for future work.

Chemical space diversity was assessed by comparing pairwise TC distributions from molecules generated by *e* MolFrag→ *e* Synth and *e* MolFrag2 → *e* Synth2. We summarized these distributions using variance, entropy, Kullback–Leibler divergence,^23^ and standard statistical tests including Bartlett’s and Kolmogorov–Smirnov tests.^24–26^ Bootstrap resampling^27^ was used to estimate confidence intervals for the diversity metrics.

All benchmarks were run on a single node with an AMD EPYC 7742 64-core processor and 1 008 GB of RAM, running Linux (kernel 6.8.0). Fragmentation of the full DUD-E set was performed as a parallel job across 64 workers.

### Fragment Classification

Fragment classification in *e* MolFrag was inconsistent for small fragments. Across the DUD-E benchmark, *e* MolFrag misclassified 2,419 free atoms as bricks and 30,752 two- or three-atom linkers as bricks. By contrast, *e* MolFrag2 labels free atoms explicitly and uses graph topology to combine adjacent linkers prior to output. The two tools produce broadly similar brick counts because both derive cuts from BRICS, but they differ substantially for linkers and free atoms: *e* MolFrag2 produced 40,730 linkers and 18,551 free atoms, whereas the original classification implied 45,306 non free-atom linkers and 39,802 free atoms after reclassification. These results indicate that *e* MolFrag2 preserves the intended brick/linker distinction more consistently while reducing unnecessary isolated free atoms, as shown in Figures 3 and 4.

**Figure 3:**
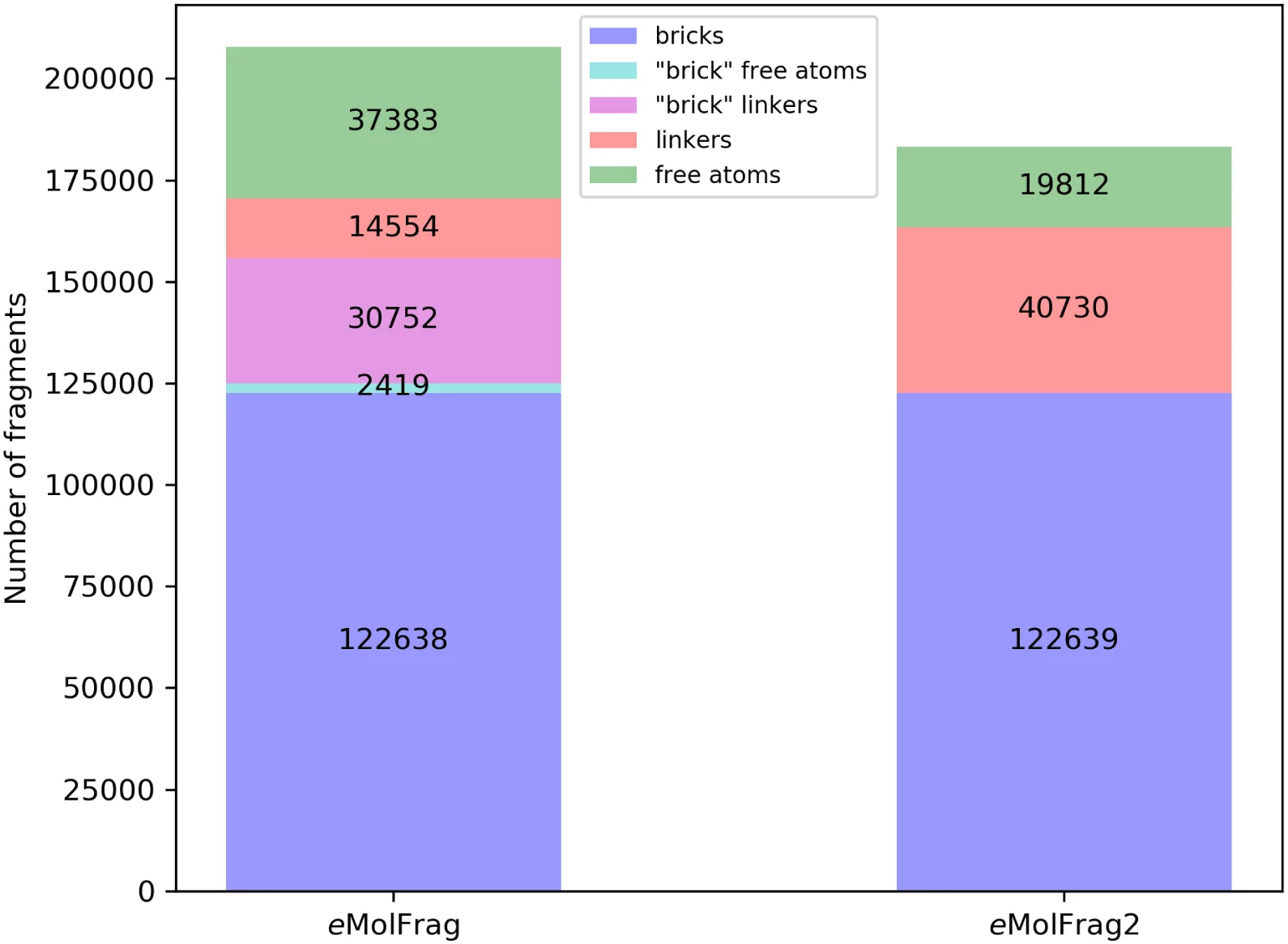
Fragment classifications from the DUD-E benchmark, produced by *e* MolFrag and *e* MolFrag2. The updated implementation explicitly separates bricks, linkers, and free atoms, reducing inconsistent assignment of small connective fragments.

**Figure 4:**
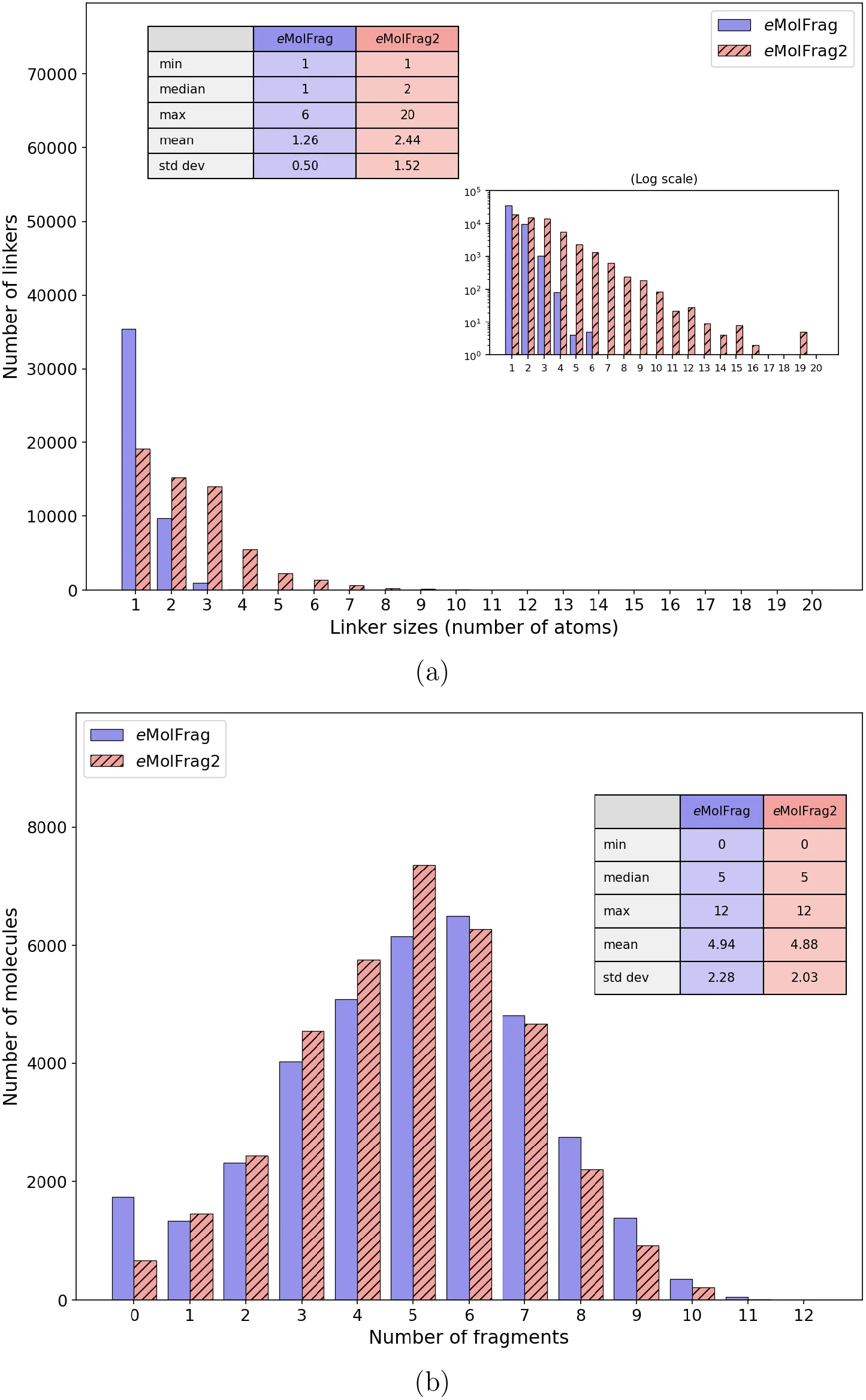
Fragmentation behavior in the DUD-E benchmark: (a) linker size distributions by atom count for *e* MolFrag and *e* MolFrag2; (b) total number of fragments produced per compound.

We compared per-fragment characteristics across five methods on test-set100 (Table S1; Figure S3 shows linker sizes and fragment counts across these methods). Because BRICS, RECAP, and MMPA do not separate bricks from linkers, we applied the *e* MolFrag2 classification uniformly. *e* MolFrag2 produces larger but fewer fragments than *e* MolFrag (6.20 versus 5.42 heavy atoms; 4.78 versus 5.11 fragments per molecule), consistent with linker combination (Table S1). BRICS closely tracks *e* MolFrag, while RECAP and MMPA yield larger fragments; MMPA’s high fragment count reflects its enumeration of multiple bond-cut combinations rather than a single decomposition. The fragment-type breakdown (Table S2) shows *e* MolFrag2 again yielding fewer linkers and free atoms than *e* MolFrag, mirroring the DUD-E result in Figure 3.

Fragmentation time reflects the additional processing in *e* MolFrag2. On the full DUD-E set, *e* MolFrag2 averaged 3.26 s per molecule versus 1.53 s for *e* MolFrag (Table S3); the additional time goes to combining adjacent linkers and recording the connectivity metadata that enables sound reconstruction. BRICS, RECAP, and MMPA fragment in roughly 0.01 s per molecule, but they do not produce the connectivity metadata that *e* MolFrag2 uses to guarantee sound reconstruction; therefore, their speed reflects a simpler task. Fragmentation is a one-time preprocessing step and parallelizes across molecules; with 64 workers, fragmentation of the full DUD-E set took well under an hour of elapsed time.

### Soundness Validation

The original *e* MolFrag→ *e* Synth workflow reconstructed exactly 51 of 100 molecules from test-set100. This rate was lower than expected from the original publications, likely because the *e* Synth publication used hand-curated fragments and because the original construction tool pruned the generated spaces with probability and molecular-property filters. Replacing only *e* Synth with *e* Synth2 improved exact reconstruction to 95 of 100 molecules. This improvement is attributable to several changes in *e* Synth2: the removal of probability-based space pruning, the removal of hard-coded Lipinski’s property filters, and the addition of support for multiple BRICS-derived bonds on a single brick atom. Together, these changes allowed *e* Synth2 to enumerate a more complete recombinant space from the same *e* MolFrag fragments. The five remaining failures were traced to *e* MolFrag aromatic-bond metadata, which stored generic aromatic bonds rather than explicit single and double bonds needed for unambiguous reconstruction.

The full updated *e* MolFrag2 → *e* Synth2 workflow reconstructed all 36,534 DUD-E compounds exactly. Twenty-three compounds required merged superfragment handling, confirming that the superfragment representation is not merely cosmetic but necessary for complete soundness on chemically diverse inputs. This result is the central validation of the application note: *e* MolFrag2 and *e* Synth2 together provide a sound fragment-and-reconstruct workflow for a large benchmark set.

We compared two reconstruction workflows on test-set100 at *TC* = 1.0. The first fragmented each molecule with BRICS.BRICSDecompose­() and rebuilt it with BRICS.BRICSBuild­(). The second fragmented with *e* MolFrag2 and rebuilt with *e* Synth2 with the unique-build option. *e* MolFrag2 → *e* Synth2 recovered all 100 molecules exactly; BRICSBuild recovered 46. Because *e* MolFrag2 emits the complete fragment set with connectivity metadata and *e* Synth2 recombines it at compatible connection points, the original molecule is contained in the generated space by construction, a guarantee BRICSBuild lacks. BRICS also produced more fragments per molecule (5.27 versus 4.78) and smaller linkers (2.35 versus 2.50 non-hydrogen atoms), consistent with *e* MolFrag2 combining adjacent linkers; the additional BRICS fragments enlarge the combinatorial space and reduce exact recovery.

RECAP and MMPA are excluded from the reconstruction comparison: RECAP provides no native reconstruction routine, and MMPA is a matched-pair analysis method that is not designed to rebuild whole molecules from fragments. We therefore compare reconstruction only to BRICS, the sole alternative with a native build routine.

### Reconstruction Throughput

*e* Synth2 operates primarily as an enumerator of a recombinant space. The relevant performance question is not whether it reconstructs a molecule faster than *e* Synth but whether removing the probability and Lipinski filters degrades generation throughput. We measured throughput using a shared pool of 20 fragments at −hl = 10. The two tools emit molecules at comparable rates: roughly 220 raw molecules per second and 204 to 209 unique, valid molecules per second (distinct RDKit canonical SMILES), differing by only a few percent. *e* Synth2 yields slightly more duplicate and invalid candidates than *e* Synth, as expected without the filters, but its net unique-valid rate is unchanged within run-to-run variation. Output grows linearly over the full run (Figure S4 and Figure S5), so the modifications impose no throughput decay or generation cost penalty. Unique-valid counts were obtained by canonicalizing and sanitizing the raw SMILES output with RDKit as a downstream step; *e* Synth2 does not itself deduplicate or validate its output. Consistent with the design principle of leaving property filtering to the user, we treat this as a postprocessing step rather than a built-in stage and recommend it for users requiring deduplicated, valid-only output. Soundness guarantees that each original molecule appears among its recombinants; it does not guarantee that every recombinant is chemically valid. Recombinants intended as training data for generative models should, therefore, pass through this sanitization step.

### Chemical Space Diversity

To evaluate the diversity of recombinant chemical spaces generated by each workflow, we conducted two analyses: a detailed examination of a single molecule (CHEMBL222590) and a combined analysis of four distinct molecules to test whether findings generalize across varied structures. CHEMBL222590 was selected for the single-molecule analysis because it violates one Lipinski Rule of Five criterion: it has 11 hydrogen-bond acceptors, exceeding the limit of 10. Its molecular weight of 497.56 Da also lies just below the 500 Da threshold. As a result, the hard-coded property filters in *e* Synth may have excluded this compound, and recombinants like it, from the generated space. *e* Synth2, with those filters removed, handles such compounds without restriction, making CHEMBL222590 an informative case for comparing the diversity of recombinant spaces produced by the two workflows. For CHEMBL222590 (Figure 5a), both workflows produced narrow similarity distributions, but *e* MolFrag2 → *e* Synth2 had higher entropy than *e* MolFrag→ *e* Synth (2.35 versus 2.11) and a Kullback–Leibler divergence of 12.5930, indicating distinct output distributions.

**Figure 5:**
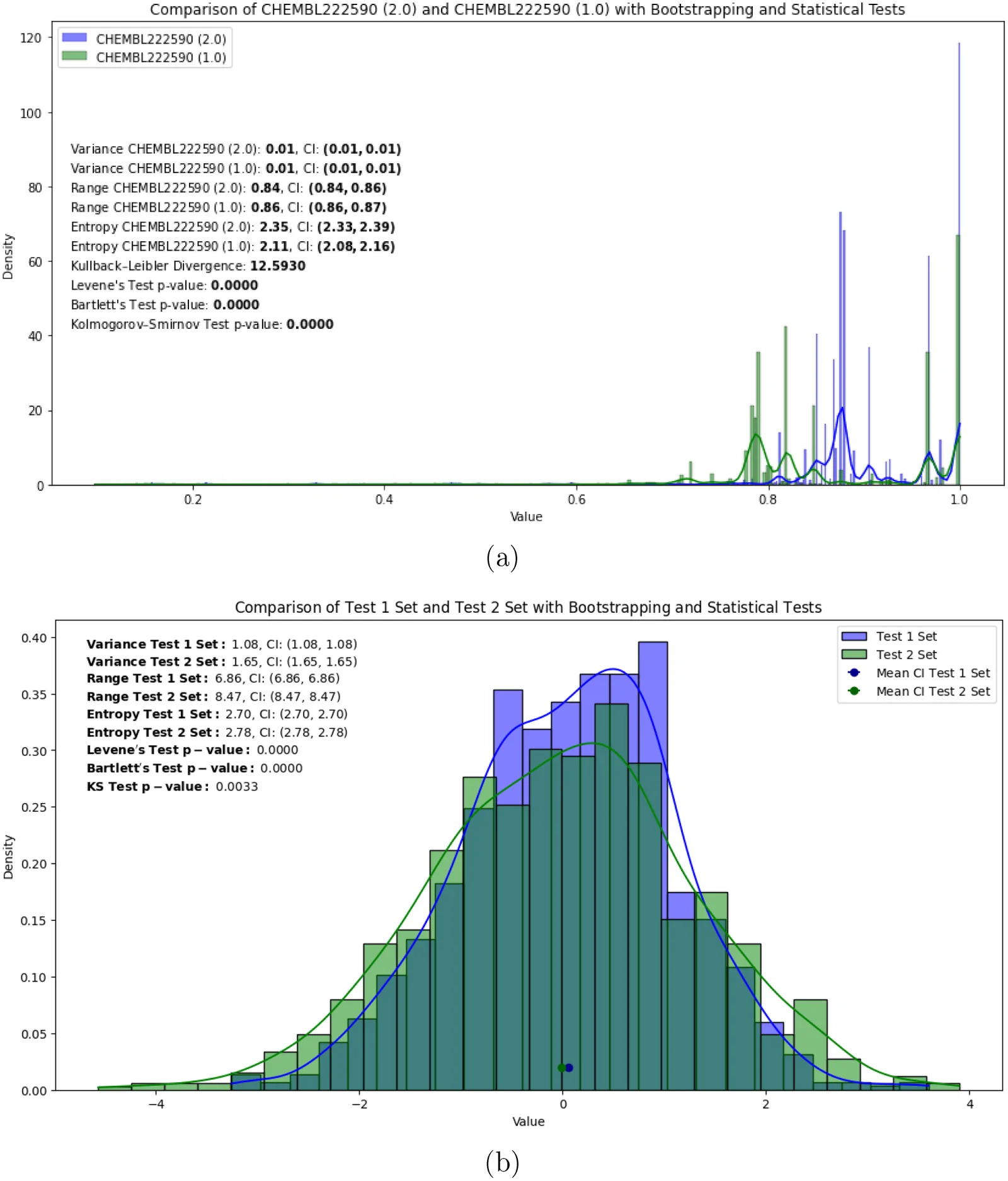
Comparative analysis of recombinant output samples generated by *e* MolFrag→ *e* Synth and *e* MolFrag2 → *e* Synth2: (a) CHEMBL222590; (b) a four-molecule sample from the DUD-E benchmark.

In a four-molecule combined analysis (Figure 5(b)), *e* MolFrag2 → *e* Synth2 produced higher variance (1.65 versus 1.08) and higher entropy (2.78 versus 2.70) than *e* MolFrag→ *e* Synth. While the entropy difference is modest in isolation, Bartlett’s test (*p* < 0.0001) and the Kolmogorov–Smirnov test (*p* = 0.0033) corroborate that the two workflows produce meaningfully distinct output distributions. These statistical measures should be interpreted together with the entropy and variance results rather than individually. These findings suggest that the updated handling of free atoms, linkers, and connectivity broadens the generated chemical space without compromising reconstruction soundness.

The practical utility of the *e* MolFrag2 → *e* Synth2 workflow is further demonstrated in a recent application by our group, in which the workflow was used to systematically explore antibiotic chemical space, reconstruct penicillin derivatives, and design hybrid molecules combining functional groups from different antibiotic classes.^28^

## Conclusions

*e* MolFrag2 and *e* Synth2 provide an updated, open-source workflow for fragment-based molecular decomposition and reconstruction. The most important advance is sound reconstruction across all 36,534 tested DUD-E active compounds, demonstrating that molecules fragmented by *e* MolFrag2 can be recovered exactly by *e* Synth2. This reliability addresses a central limitation of the original *e* MolFrag→ *e* Synth workflow and makes the updated tools better suited for reproducible chemical space exploration.

The improvements are also practical for users. *e* MolFrag2 adds graph-based fragmentation, explicit free-atom and radical-atom handling, linker combination, superfragment support, and unambiguous aromaticity metadata. *e* Synth2 uses this richer fragment information to support more general reconstruction, including multiple bonds to a single brick atom and targeted soundness validation without hard-coded property filters. Together, these changes preserve chemically meaningful connection points while giving researchers more control over the recombinant spaces they generate.

We expect the *e* MolFrag2 → *e* Synth2 workflow to be useful for fragment-based drug discovery^29,30^ virtual screening library construction and benchmarking studies that require transparent and reproducible molecular fragmentation and reconstruction. Beyond the applications demonstrated here, we anticipate that the soundness guarantee provided by *e* MolFrag2 → *e* Synth2 will be of particular value in emerging AI-driven workflows. Fragment libraries produced by *e* MolFrag2 can serve as structured inputs for generative molecular design models,^7^ where sound fragmentation ensures that the fragments supplied to the model carry complete and unambiguous connectivity information. As fragment-based approaches become increasingly integrated with machine learning pipelines, tools that guarantee sound decomposition and reconstruction will be essential for producing reproducible and interpretable results.

## Supplementary Material



## Data Availability

The source code for *e*MolFrag2 and *e*Synth2 is publicly available under the MIT License at https://github.com/deepdrugai/eMolFrag2 and https://github.com/deepdrugai/eSynth2, respectively. Benchmark validation used active compounds from the DUD-E database.^12^ No proprietary or confidential data were used in this work.

## Notes

A worked example of superfragment construction for the DUD-E molecule ALDR-102 (Figure S1); quantile–quantile plot comparing fragment-count distributions for test-set100 and the full DUD-E active set (Figure S2); per-fragment characteristics of five fragmentation methods on test-set100 (Table S1); linker-size and fragment-count distributions of five methods (Figure S3); fragment-type breakdown of five methods (Table S2); fragmentation timing of five methods on the full DUD-E active set (Table S3); reconstruction throughput of *e*Synth and *e*Synth2, raw and unique-valid molecule generation (Figures S4 and S5) (PDF)

## References

[1] Hajduk P. J., Greer J. (2007). A decade of fragment-based drug design: strategic advances and lessons
learned. Nat. Rev. Drug Discovery.

[2] Lewell X. Q., Judd D. B., Watson S. P., Hann M. M. (1998). RECAP–Retrosynthetic
Combinatorial Analysis Procedure: A Powerful New Technique for Identifying
Privileged Molecular Fragments with Useful Applications in Combinatorial
Chemistry. J. Chem. Inf. Comput. Sci..

[3] Degen J., Wegscheid-Gerlach C., Zaliani A., Rarey M. (2008). On the art of compiling
and using ‘drug-like’ chemical fragment spaces. ChemMedChem.

[4] Hussain J., Rea C. (2010). Computationally Efficient Algorithm to Identify Matched Molecular
Pairs (MMPs) in Large Data Sets. J. Chem. Inf.
Model..

[5] Walters W.
P., Stahl M. T., Murcko M. A. (1998). Virtual screening—an overview. Drug Discovery Today.

[6] Schuffenhauer A. (2012). Computational
methods for scaffold hopping. WIREs Comput.
Mol. Sci..

[7] Meyers J., Fabian B., Brown N. (2021). De novo molecular
design and generative
models. Drug Discovery Today.

[8] Podda, M. ; Bacciu, D. ; Micheli, A. A Deep Generative Model for Fragment-Based Molecule Generation arXiv 2020 10.48550/arXiv.2002.12826

[9] Liu T., Naderi M., Alvin C., Mukhopadhyay S., Brylinski M. (2017). Break Down in Order To Build Up: Decomposing Small
Molecules for Fragment-Based Drug Design with eMolFrag. J. Chem. Inf. Model..

[10] Naderi M., Alvin C., Ding Y., Mukhopadhyay S., Brylinski M. (2016). A graph-based approach to construct target-focused
libraries for virtual screening. J. Cheminform..

[11] Landrum, G. RDKit: open-Source Cheminformatics; 2006. https://www.rdkit.org.

[12] Mysinger M. M., Carchia M., Irwin J. J., Shoichet B. K. (2012). Directory
of Useful
Decoys, Enhanced (DUD-E): Better Ligands and Decoys for Better Benchmarking. J. Med. Chem..

[13] Hagberg, A. A. ; Swart, P. J. ; Schult, D. A. Exploring Network Structure, Dynamics, and Function Using NetworkX. In Proceedings of the 7th Python in Science Conference (SciPy 2008); Pasadena, CA, USA, 2008; pp. 11–15.

[14] Wishart D. S., Feunang Y. D., Guo A. C., Lo E. J., Marcu A., Grant J. R., Sajed T., Johnson D., Li C., Sayeeda Z. (2018). Drugbank 5.0: A major update to the DrugBank
database
for 2018. Nucleic Acids Res..

[15] Weininger D., Weininger A., Weininger J. L. (1989). SMILES. 2. Algorithm for generation
of unique SMILES notation. J. Chem. Inf. Comput.
Sci..

[16] O’boyle N. M., Banck M., James C. A., Morley C., Vandermeersch T., Hutchison G. R. (2011). Open Babel: An open chemical toolbox. J. Cheminf..

[17] Lipinski C. A., Lombardo F., Dominy B. W., Feeney P. J. (2001). Experimental and
computational approaches to estimate solubility and permeability in
drug discovery and development settings. Adv.
Drug Delivery Rev..

[18] Hosseini M., Pereira D. M. (2023). The Chemical Space of Terpenes: Insights
from Data
Science and AI. Pharmaceuticals.

[19] Paduch R., Kandefer-Szerszeń M., Trytek M., Fiedurek J. (2007). Terpenes:
substances useful in human healthcare. Arch.
Immunol. Ther. Exp..

[20] Wilk M. B., Gnanadesikan R. (1968). Probability plotting methods for
the analysis for the
analysis of data. Biometrika.

[21] Tanimoto, T. T. An elementary mathematical theory of classification and prediction; 1958.

[22] Bajusz D., Rácz A., Héberger K. (2015). Why is Tanimoto index an appropriate
choice for fingerprint-based similarity calculations?. J. Cheminform..

[23] Kullback S., Leibler R. A. (1951). On Information and
Sufficiency. Ann. Math. Stat..

[24] Bartlett M. S. (1937). Properties
of Sufficiency and Statistical Tests. Proc.
R. Soc. London, Ser. A.

[25] Kolmogorov A. N. (1933). Sulla determinazione
empirica di una legge di distribuzione. G. dell’Istituto
Ital. Attuari.

[26] Smirnov N. (1948). Table for
estimating the goodness of fit of empirical distributions. Ann. Math. Stat..

[27] Mooney, C. Z. ; Duval, R. D. ; Duvall, R. Bootstrapping: A nonparametric approach to statistical inference; sage, 1993.

[28] Bess A., Rowland S., Alvin C., Mukhopadhyay S. (2026). AI Derivation
and Exploration of Antibiotic Class Spaces. J. Cheminform..

[29] Ranjan A., Bess A., Alvin C., Mukhopadhyay S. (2024). MDF-DTA: a
multi-dimensional fusion approach for drug-target binding affinity
prediction. J. Chem. Inf. Model..

[30] Bess A., Berglind F., Mukhopadhyay S., Brylinski M., Griggs N., Cho T., Galliano C., Wasan K. M. (2022). Artificial
intelligence for the discovery of novel antimicrobial agents for emerging
infectious diseases. Drug Discovery Today.

