# Supplementary material for: e MolFrag2 and e Synth2: Sound Fragment-Based Molecular Decomposition and Reconstruction
Source: J Chem Inf Model. 2026 Oct 13;66(20):13023–31. doi: 10.1021/acs.jcim.6c01714 (PMC13715516; doi:10.1021/acs.jcim.6c01714)
Supplement: Supplementary file 1 [file ci6c01714_si_001.pdf]

Supporting Information for

*e*MolFrag2 and *e*Synth2: Sound Fragment-Based Molecular Decomposition and Reconstruction

Adam Bess,[1] Sean Rowland,[2] Chris Alvin,[2] Meisam Adibifard,[3] and Supratik Mukhopadhyay[3]

[1]Department of Computer Science, Louisiana State University, Baton Rouge, LA, USA-70803
[2]Department of Computer Science, Furman University, Greenville, SC, USA-29613
[3]Department of Environmental Sciences, Louisiana State University, Baton Rouge, LA, USA-70803

Corresponding author: chris.alvin@furman.edu

Worked example of super-fragment construction

Consider aldr-102, depicted in Figure S1, which *e*`MolFrag2` decomposes into exactly 6 fragments: 3 brick carbon rings with pairwise $TC = 1.0$, a unique brick, and 2 linkers with $TC = 1.0$. As shown in Figure S1(b), brick 003 has the richest connectivity information among the carbon rings because it can bond with `C.2` at atom 1 and `N.am` at atom 4. During construction with *e*`Synth2`, if fragment 000 (or fragment 001) is chosen to connect to another molecule r, yielding the SMILES representation $smi_{r+f_{000}}$, then combining fragment 003 with the same molecule r produces $smi_{r+f_{003}}$, which is equivalent to $smi_{r+f_{000}}$. Without super-fragment handling, $r + f_{003}$ would be filtered as redundant and reconstruction with $TC = 1.0$ would fail. The aldr-102 example illustrates this case: chemically identical ring fragments have different connection sites, and the super-fragment representation prevents redundant reconstruction while preserving all valid connection patterns.

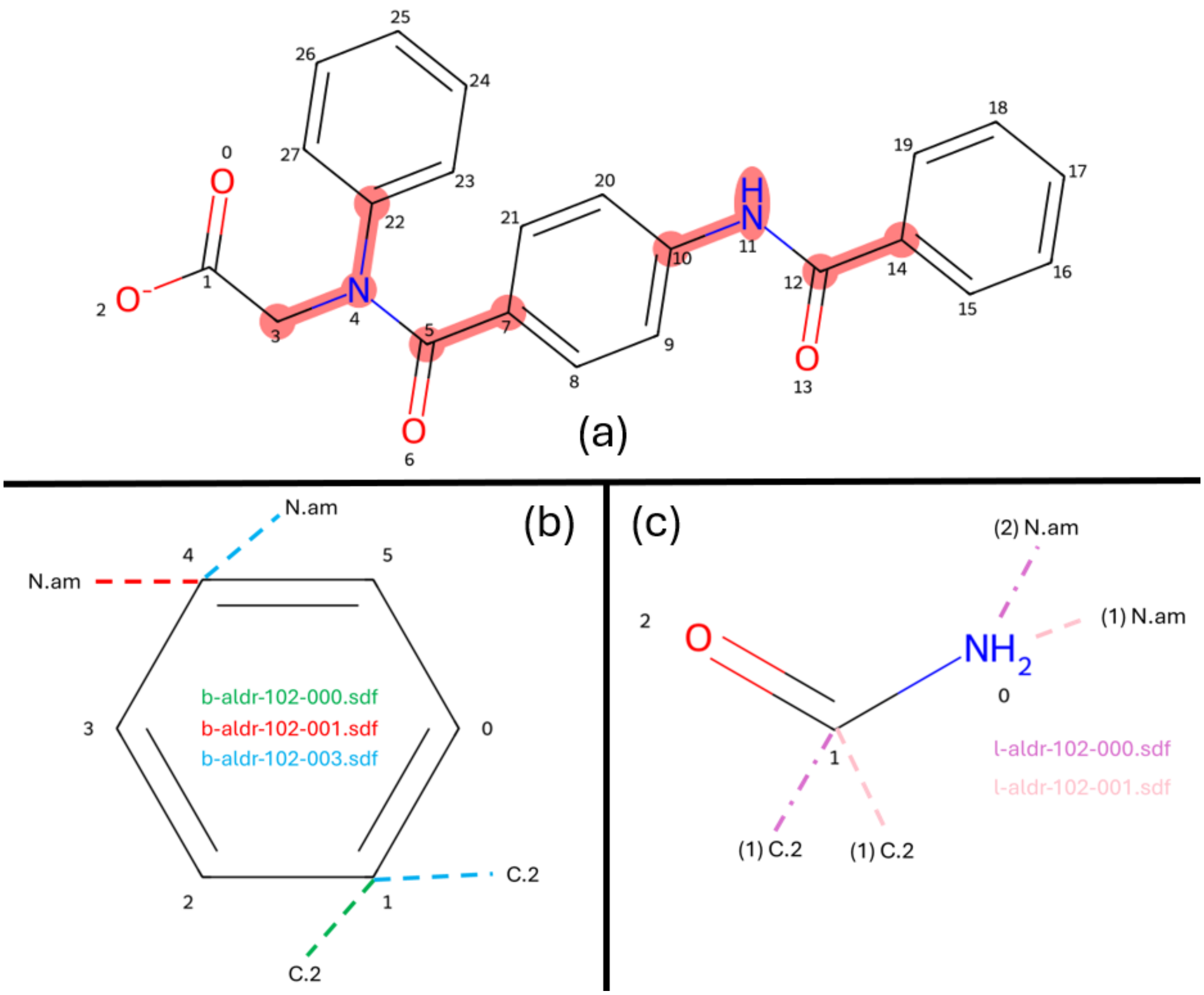

Figure S1: (a) DUD-E molecule aldr-102 with the bonds cut by *e*`MolFrag2` highlighted, producing exactly 6 fragments: 3 brick carbon rings with pairwise $TC = 1.0$, a unique brick, and 2 linkers with $TC = 1.0$. (b) The super-fragment for the 3 brick carbon rings; brick 003 encompasses all connection points of bricks 000 and 001. (c) The super-fragment for the 2 linkers; linker 000 exposes two `N.am` connection points while linker 001 exposes only one, making linker 000 the super-fragment.

Representativeness of `test-set100`

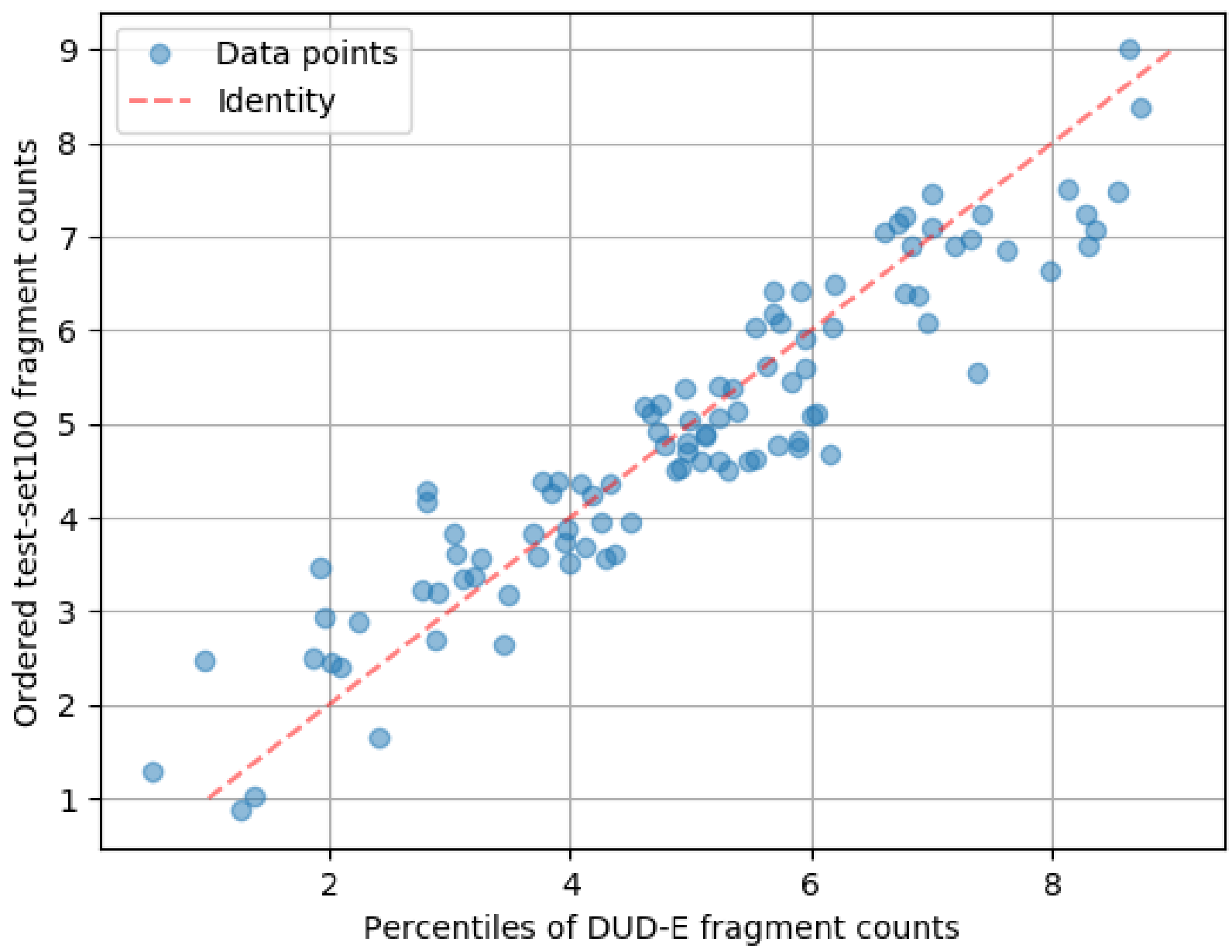

Figure S2: Quantile–quantile (Q–Q) plot comparing the distribution of total fragment counts between `test-set100` and all 36,534 DUD-E active compounds. A jitter value of 0.5 is added for readability because both distributions are discrete counts of constituent fragments.

Comparison of fragmentation methods

Table S1: Per-fragment characteristics on `test-set100` for five fragmentation methods. Values are mean ± standard deviation. Heavy atom, heteroatom, ring, and linker-size values are per fragment; fragment count is per molecule. A common classification is applied across all methods (free atoms: one non-hydrogen atom; linkers: two or three; bricks: four or more). Fragment counts are not directly comparable across methods: RECAP and especially MMPA enumerate multiple bond-cut combinations per molecule rather than a single decomposition, which inflates their fragment counts and fragment sizes.

Method	Heavy atoms	Heteroatoms	Rings	Fragment count	Linker size
e`MolFrag`	5.42 ± 4.41	1.37 ± 1.28	0.70 ± 0.86	5.11 ± 1.83	2.30 ± 0.46
e`MolFrag2`	6.20 ± 4.17	1.54 ± 1.37	0.81 ± 0.86	4.78 ± 1.61	2.50 ± 0.50
BRICS	5.41 ± 4.26	1.33 ± 1.33	0.71 ± 0.84	5.27 ± 1.81	2.35 ± 0.48
RECAP	8.92 ± 5.77	2.07 ± 1.64	1.17 ± 0.92	3.21 ± 1.40	2.44 ± 0.51
MMPA	15.99 ± 10.59	3.96 ± 3.05	2.00 ± 1.54	14.59 ± 5.45	2.56 ± 0.50

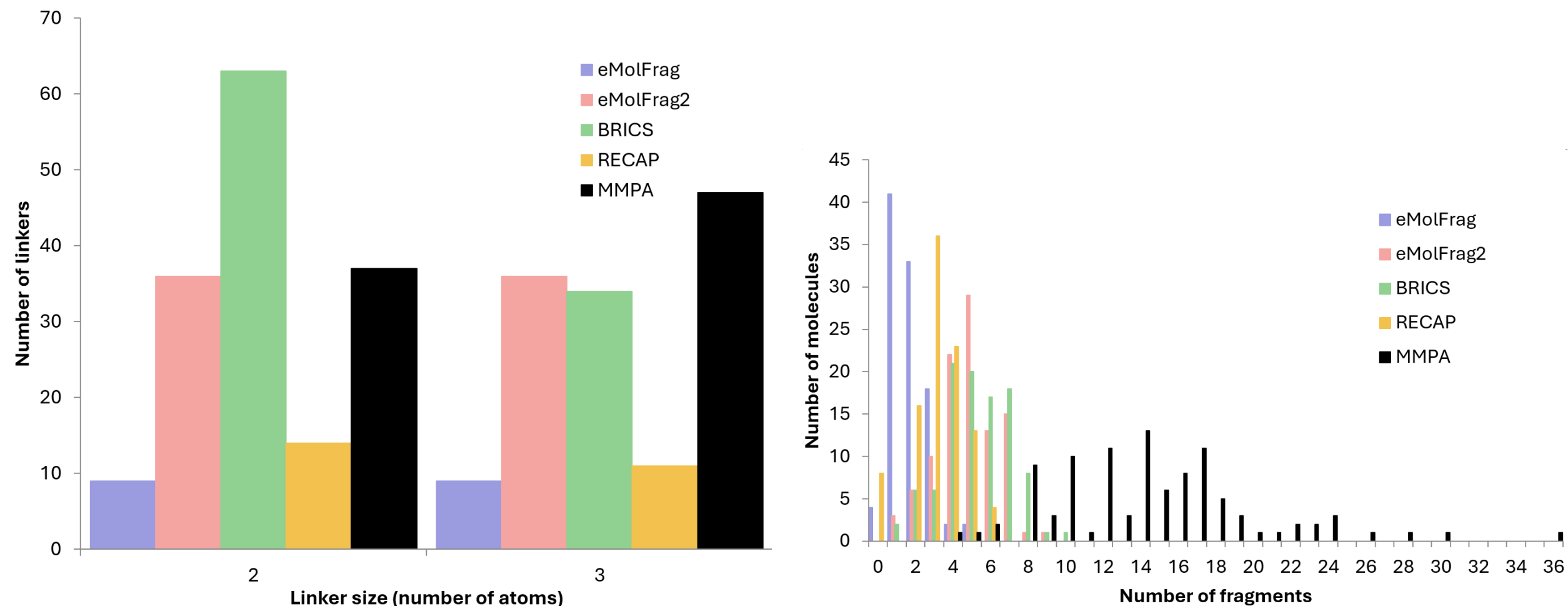

(a) (b)

Figure S3: Fragmentation behavior on `test-set100` across five methods (*e*`MolFrag`, *e*`MolFrag2`, BRICS, RECAP, and MMPA): (a) linker size distribution by atom count; (b) number of fragments produced per molecule. MMPA's broad fragment-count distribution reflects its enumeration of multiple bond-cut combinations per molecule rather than a single decomposition.

Table S2: Fragment-type breakdown on `test-set100` for five fragmentation methods. Counts are totals over the 100 molecules, using a uniform classification (free atoms: one non-hydrogen atom; linkers: two or three; bricks: four or more). RECAP and MMPA use different cut rules from the BRICS-based methods, and MMPA enumerates multiple bond-cut combinations per molecule, which inflates its totals.

Method	Bricks	Linkers	Free atoms	Total
e`MolFrag`	307	109	95	511
e`MolFrag2`	362	72	44	478
BRICS	326	97	104	527
RECAP	278	25	18	321
MMPA	1243	84	132	1459

Table S3: Fragmentation time on the full DUD-E active set (36,534 compounds), run as a 64-worker parallel job on an AMD EPYC 7742. Time per molecule is the mean ± standard deviation of single-core processing time. Aggregate core-time is the summed processing time across all molecules; elapsed wall-clock is approximately this value divided by the worker count. *e*`MolFrag` and *e*`MolFrag2` perform graph-based processing that yields the connectivity metadata and combined linkers required for sound reconstruction; BRICS, RECAP, and MMPA are lightweight decompositions that do not.

Method	Time per molecule (s)	Aggregate core-time (s)
e`MolFrag`	1.53 ± 0.33	55,871.76
e`MolFrag2`	3.26 ± 0.66	119,089.02
BRICS	0.01 ± 0.01	289.82
RECAP	0.01 ± 0.01	258.63
MMPA	0.01 ± 0.01	361.39

Reconstruction throughput

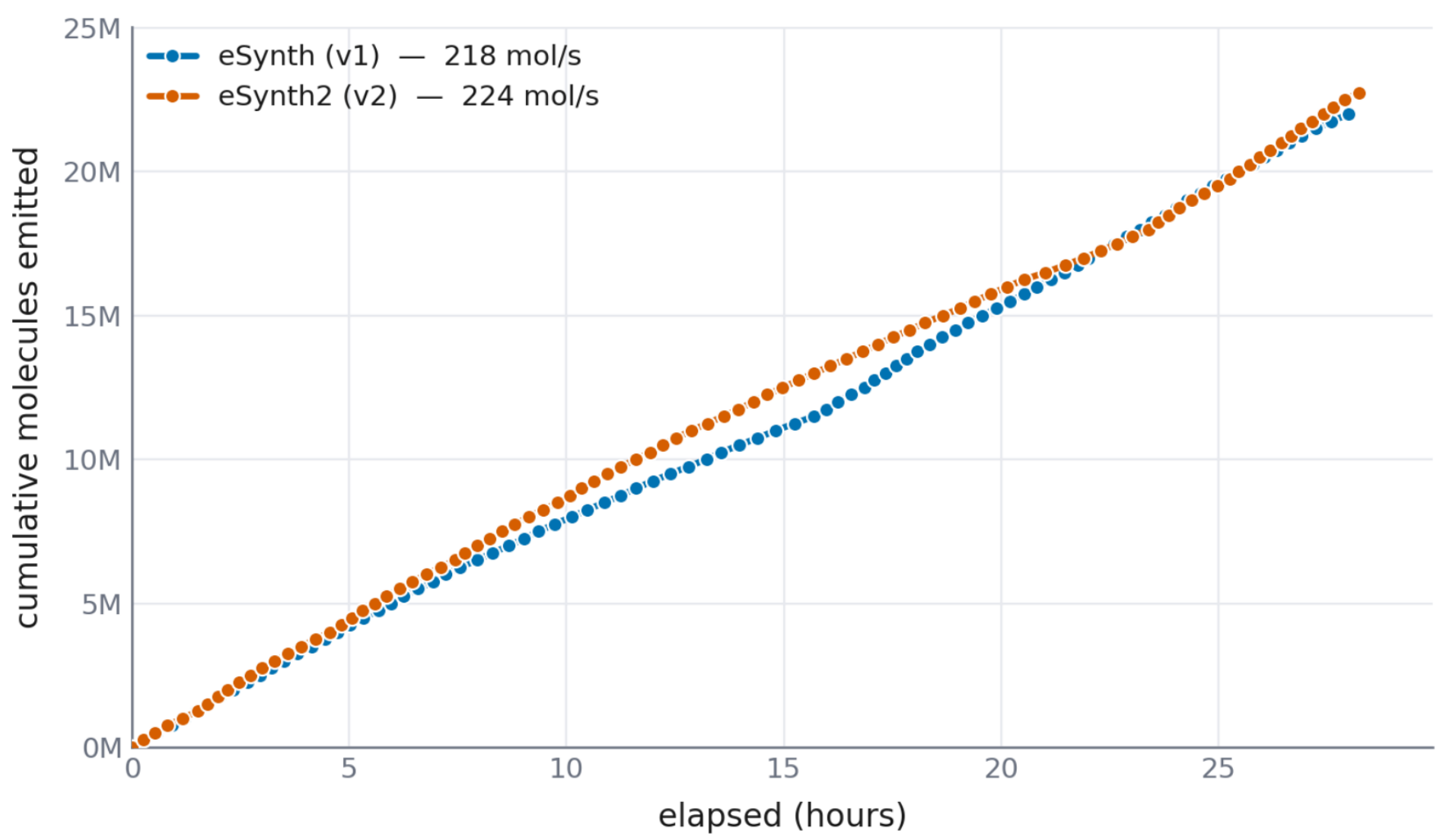

Figure S4: Cumulative molecules emitted versus wall-clock time for *e*`Synth` and *e*`Synth2` on a shared fragment pool (20 fragments, `-hl` = 10). Markers denote completion of successive 250,000-molecule chunks. Raw enumeration throughput is comparable between the two tools (*e*`Synth`, 218 molecules per second; *e*`Synth2`, 224 molecules per second), and cumulative output is linear throughout, indicating no throughput decay. Rates in the legend are given in molecules per second.

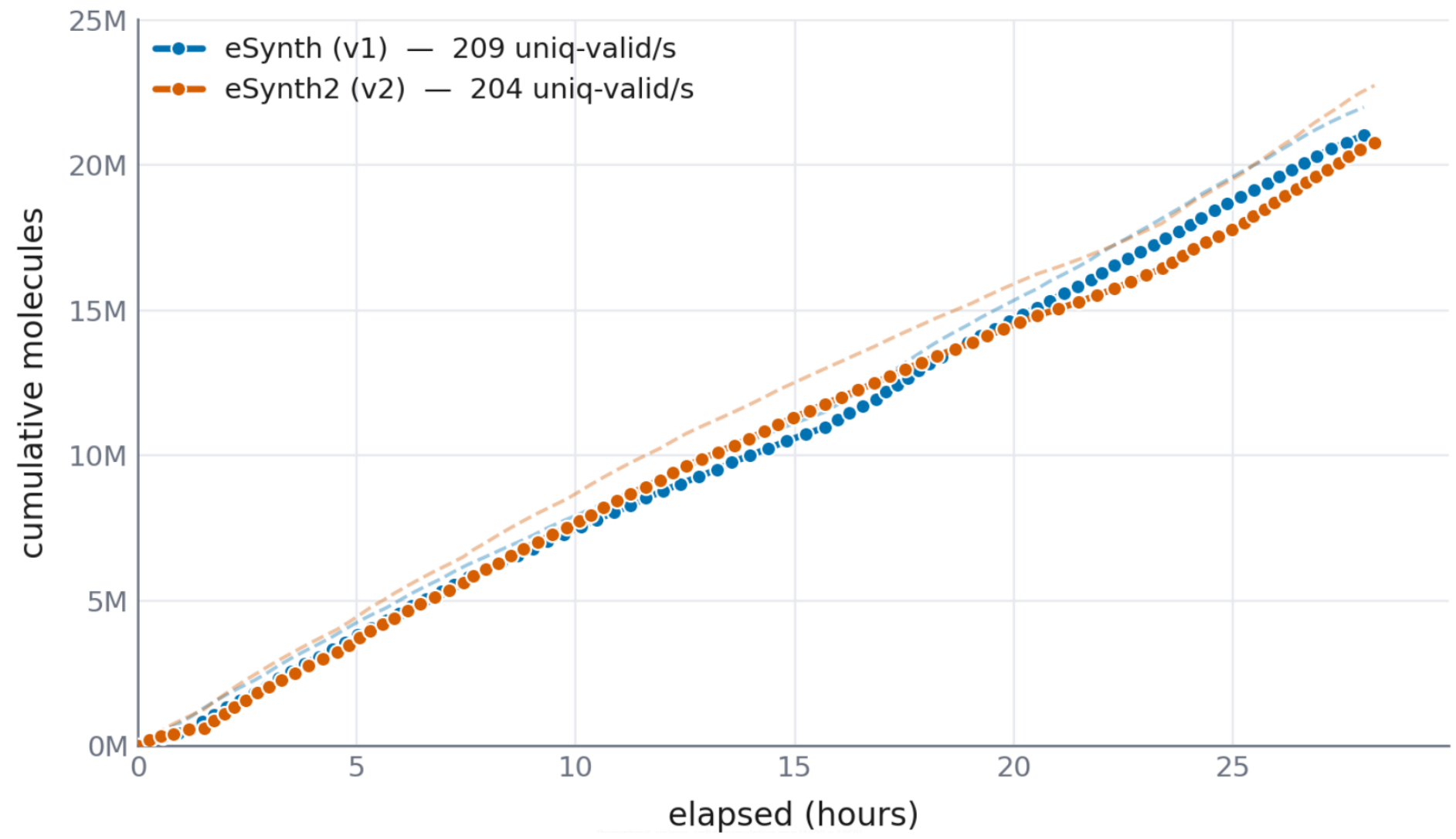

Figure S5: Cumulative unique, valid molecules (distinct RDKit canonical SMILES; solid) versus raw output (dashed) for *e*`Synth` and *e*`Synth2`; the gap between the curves reflects duplicate and invalid candidates. The rate of unique, valid molecules is comparable between the tools (*e*`Synth`, 209 molecules per second; *e*`Synth2`, 204 molecules per second). *e*`Synth2` shows a marginally wider gap, consistent with its removal of the pre-construction filters. Rates in the legend are given in molecules per second.
